# Application of Edge Computing Technology in Hydrological Spatial Analysis and Ecological Planning

**DOI:** 10.3390/ijerph18168382

**Published:** 2021-08-08

**Authors:** Xinhong Cai, Dawei Xu

**Affiliations:** 1 College of Landscape Architecture, Northeast Forestry University, Harbin 150000, China; xinhongcai96@gmail.com; 2 Key Lab for Garden Plant Germplasm Development, Landscape Eco-Restoration in Cold Regions of Heilongjiang Province, Harbin 150000, China

**Keywords:** edge computing, intelligent environmental protection, hydrological spatial analysis, ecological planning

## Abstract

The process of rapid urbanization causes so many water security issues such as urban waterlogging, environmental water pollution, water shortages, etc. It is, therefore, necessary for us to integrate a variety of theories, methods, measures, and means to conduct ecological problem diagnosis, ecological function demand assessment, and ecological security pattern planning. Here, EC (Edge Computing) technology is applied to analyze the hydrological spatial structure characteristics and ecological planning method of waterfront green space. First, various information is collected and scientifically analyzed around the core element of ecological planning: water. Then, in-depth research is conducted on the previous hydrological spatial analysis methods to identify their defects. Subsequently, given these defects, the EC technology is introduced to design a bottom-up overall architecture of intelligent ecological planning gateway, which can be divided into field devices, EC intelligent planning gateway, transmission system, and cloud processing platform. Finally, the performance of the overall architecture of the intelligent ecological planning gateway is tested. The study aims to optimize the performance of the hydrological spatial analysis method and ecological planning method in Xianglan town of Jiamusi city. The results show that the system can detect the flood control safety system planning, analysis of water source pollution. Additionally, the system also can use the EC technology, depending on the types, hydrological characteristics, pollutants to predict treatment sludge need to put in the pollutant treatment medicament composition and dosage, protection of water source nearby residents public health security. Compared with previous hydrological spatial analysis and ecological planning methods, the system is more scientific, efficient, and expandable. The results provide a technical basis for the research in related fields.

## 1. Introduction

Water is essential for human existence, and human settlements are formed around water sources. Therefore, land areas surrounded by rivers, lakes, or oceans have become the best location for city construction because of convenient transportation and superior ecological environment. Meanwhile, the 21st century has witnessed the emergence and popularization of ecological civilization [1,2], with which the traditional human-centered values are being replaced by the values of ecological civilization coordinating humans and nature. In terms of ecological civilization construction, the report of the 18th CPC (Communist Party of China) National Congress has pointed out the civilized ecological concept of respecting nature, conforming to nature, and protecting nature. Since the proposal of the concept of the ecological city in the 1970s, it has been developed and enriched in urban construction around the world [3]. Significantly, waterfront green space is an important part of the construction of an ecological city, whose development and construction have shifted from a single factor human-oriented development to a dual-centered development of humans and nature. Nowadays, many landscape planners and related researchers in China and other countries are constantly studying the ecological planning of waterfront green space. Among the surface water sources in China, lake and reservoir water sources account for about 40%, which are an important type of drinking water sources in China. The results show that the main pollutants in lake and reservoir water sources in China include chemical oxygen demand (COD), total nitrogen (TN), and total phosphorus (TP), and the pollution sources are mainly point source pollution, non-point source pollution, and internal source pollution. Polluted drinking water seriously affects public health security. Edge computing (EC) refers to the end close to objects or data sources, which adopts an open platform integrating core capabilities of network, computing, storage and application to provide services nearby. In the traditional water pollutant treatment process, the problem of point-sampling analysis and point-dispensing method is that there is a large amount of treatment delay and waste of treatment resources. Specifically, Badach and Raszeja (2019) [4] pointed out that UL (Urban Landscape) management and UG (Urban Greenspace) delivery needed effective planning tools. The purpose of their research was to provide a conceptual framework for the implementation of the ecological, structural, and visual landscape, and LGI (Landscape and Greenspace Indicators) in urban spatial development. Based on the existing planning documents, the applicability of the selected LGI was considered in the Polish planning system. Further, the quality of UL and UG transformation is discussed in three case studies in Bristol, Gdańsk, and Poznań. The results showed that the implementation of the LGI framework could significantly improve the ecological quality, visual quality, and structural diversity of UL and UG. Linglan et al. (2018) [5] studied the environmental degradation of densely built areas in the urbanization process in China. In consideration of the lack of green space and open space in densely built-up areas, ecological disturbance, and poor landscape quality, the optimization of ecological space was mainly analyzed in urban renewal. Firstly, the relevant theories were analyzed, and a comprehensive ecological efficiency evaluation system was established based on urban ecology, landscape ecology, urban sociology, behavioral psychology, biology, and urban planning and design. Secondly, the ecological efficiency of typical blocks was judged by the established system on the GIS (Geographical Information System) platform, and the key spatial nodes that need to be updated were found. Thirdly, different theories of spatial optimization were designed in different cases, and the ecological efficiency of the model was evaluated comprehensively. Finally, the parameters under different conditions were modified to get a greenspace evaluation system for the built-up area. Chen et al. (2020) [6] pointed out that the state of land and resources should be considered from the foundation of China’s economic and social development, and the proposal had aroused great attention to the sustainable development of land and resources. China’s economy had developed steadily and entered a new stage, in which land and resources should be planned more reasonably to ensure sustainable economic development and long-term social stability. Therefore, the management of land and resources should be improved. The original technology was improved with scientific planning methods to protect and develop China’s land and resources reasonably and contribute to the sustainable development of China and the world. Lin et al. (2020) [7] pointed out that watershed landscapes could reflect and drive the dynamic changes of water quality along the river. Therefore, scientific landscape ecological planning is the key to flow quality management. Then, an improved and targeted ecological planning method was proposed based on landscape ecology, emphasizing the importance of CSSL (Characteristic Source-Sink Landscape) and their structure that contribute mostly to the stream quality change of mountain rivers in sustainable management for mountain stream quality. The research was conducted in the Yinglong watershed in the mountainous area of Chongqing and the landscape ecological planning was implemented through CSSL extraction—constraint functions construction—CSSL optimization. The results implied that it was feasible to optimize the main conflict landscape closely related to the change of stream quality through the landscape ecology method to help effectively manage stream quality. Meanwhile, this more targeted and measurable ecological planning method could promote the transformation of theoretical planning results into planning practice and could encourage landscape planners besides researchers to participate in specific ecological planning practices.

To sum up, there are many research achievements in ecological planning, but there are also some problems. Therefore, to handle complex site hydrological spatial information data, an effective and scientific method should be proposed with high data-processing efficiency, clearer and more accurate target capture ability, perfect logical system, and scientific quantitative analysis ability for hydrological space and waterfront green space. Therefore, the study takes Xianglan town of Jiamusi city as an example, applies EC technology in hydrological spatial analysis and ecological planning, and puts forward a complete optimization system.

## 2. Materials and Methods

### 2.1. Hydrology and Hydrological Characteristics

Hydrology is a natural science to study the change and movement, the law of space-time change, and mutual transformation of the natural water system. From the perspective of geography, hydrology focuses on the performance of nature and provides scientific references for human production and life based on the study of nature’s laws. In the past, hydrology mainly considers hydrological circulation water volume in nature using physical, mathematical, and statistical methods. Hence, it is widely applied in flood forecasts. Yet, the demand for a water environment is increasing with expanding human settlements. Meanwhile, natural disasters, such as floods and rainstorms, as well as the destruction of the ecological system and human-induced water pollution, have severely obstructed socio-economic development and caused life and property loss to people. Therefore, the research on solving the contradictions between the water system and humans has become crucial [8,9,10].

Contemporary hydrology utilizes modern technologies to obtain the time–space distribution and movement of water on the earth. Further, contemporary hydrology studies the transformation and change of the water on the earth and the relationship between the hydrosphere and other circles using advanced theories and methods, thereby achieving the harmonious development of man and nature. Here, the hydroelectricity space refers to the description and expression of the hydrological process and its characteristic space in the basin. The composition of hydrological space is generally determined by the factors of hydrology formation. According to the research theme here, the most direct factors affecting hydrological space are terrain factors and climate factors because the terrain can decide the direction of water flow. Additionally, the height and shape of the terrain directly impact water velocity and water flow. While climate factors, such as precipitation, affect the water volume of the basin [11]. LV and Xiu (2020) pointed out that the stability of mobile network systems was also related to the frequency of data signals [12].

**Figure 1:**
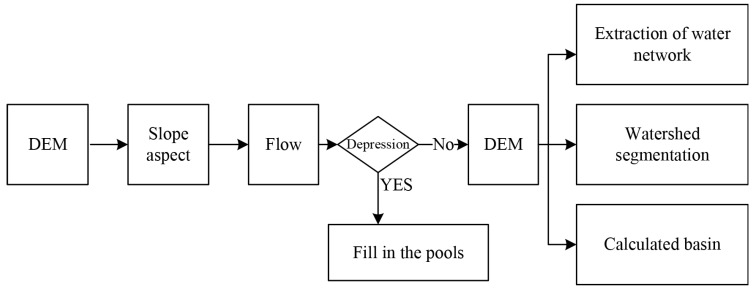
Process of hydrological spatial analysis.

With technological advancement, GIS has also prospered, and hydrological spatial analysis is the key to GIS. Hydrological analysis stimulates the surface hydrological process using the current and historical hydrological characteristics and estimates the future surface hydrological process to determine problems, such as the source seeking of surface runoff pollution, flood forecasting with intensity, and scope. DEM (Digital Elevation Model) can truly and effectively respond to the actual topography and describe basin characteristics, analyze slope aspects, and is widely used in the hydrological spatial analysis. Basic hydrological factors can be obtained from the DEM through hydrological spatial analysis and used to study the surface flow direction and basin range so that the flow process and hydrological distribution can be reproduced [13,14,15]. The specific process of hydrological spatial analysis is shown in Figure 1.

Figure 1 illustrates that the pre-processing algorithm in the process of hydrological spatial analysis is the stage of slope aspect analysis and depression filling. Once the pre-processing algorithm is completed, the DEM generation algorithm, flow direction analysis algorithm, and extraction of water network algorithm can be used for basin calculation and watershed segmentation.

### 2.2. Parallel Computing and EC

**Figure 2:**
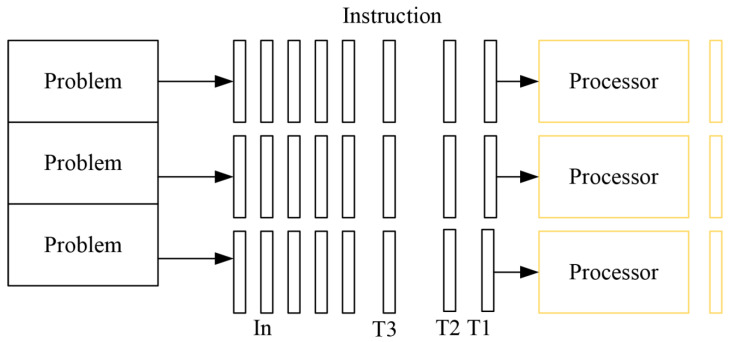
Parallel computing.

The essence of parallel computing is to use multiple computing resources to solve a computing problem simultaneously, which decomposes a problem into discrete parts to be executed concurrently. Each discrete part can be further decomposed into discrete instructions, and instructions can be executed on different processors concurrently. An overall control/cooperation mechanism schedules the execution of different parts. The schematic diagram of parallel computing is shown in Figure 2. Parallel computing has three advantages: firstly, it saves time and cost because the parallel computing system can be constructed with cheap single machines. Secondly, it can solve more large-scale and complex problems that a single machine cannot handle. Thirdly, it provides concurrency because a single computing resource process one transaction at a given time, but multiple computing resources can process multiple events concurrently [16,17,18].

With the continuous development of IoT (Internet of Things) technology and big data technology, the IoT and big data are becoming more correlated, and the data processing work has been multiplied. The forecast data of the consulting company show that 20% of the global data traffic this year is generated from mobile devices. The traditional cloud computing model can no longer adapt to the massive amount of mobile device applications with low delay and high bandwidth. Consequently, the EC model comes into being. The core of EC technology is to shift the execution center of computing tasks, that is, to unload some computing tasks from the cloud service center to the edge devices with enhanced computing power. Thus, the load of the cloud service center can be reduced, the network congestion can be alleviated, and the task processing efficiency can be improved. Particularly, the processing objects of EC are divided into the data generated by the downlink tasks from cloud services and the data generated by the uplink tasks from IoT services. In actual scenarios, EC is integrated into the distributed computing models with storage, computing, and network functions, which can process the data of the edge devices and bring DPE (data processing equipment) close to the edge of the user’s mobile network, thereby providing users with higher quality and more efficient edge intelligent services and improving service quality and customer satisfaction. IoT, mobile big data analysis, and Internet of vehicles are common application scenarios of MEC (mobile edge computing) [19,20]. Compared with traditional cloud computing technology, EC has four advantages: (1) short response time delay, (2) high security and privacy, (3) low communication overhead, and (4) strong scalability. Technologically, EC has the following four advantages: the first is isolation, edge server is usually a local machine, whose runtime will be isolated from other parts of the network, and can access local resources. The second is a low delay. Because the EC server is closer to the device terminal, it can minimize the communication delay between the terminal and the server and improve the user experience. The third is near deployment. In EC, the computing center is close to the data source. The fourth is location awareness, that is, when a part of the wireless network is at the edge, the local service can determine the location of each link device through the low-level signaling instruction under any communication conditions [21,22]. However, EC also faces many challenges, which can be divided into the following five points: (1) the traditional TCP/IP (Transmission Control Protocol/Internet Protocol) is not suitable for the current EC environment, so the design of new Internet Protocol in EC is one of the challenges. (2) How to integrate massive amounts of data is the second challenge of EC. (3) The IP (internet protocol) nodes are not enough for complex nodes in WSN (wireless sensor networks), so node-naming becomes the third challenge of EC. (4) Edge-node users are not as professional as engineers in the data center, so problems cannot be solved easily by users themselves, and they are under the threat of hackers. Thus, the protection mechanism is the fourth challenge of EC. (5) Edge nodes produce data relentlessly, while the storage capacity of edge nodes is limited, and data handling and storage become the key to the CE, thus, data management is the fifth challenge of EC.

**Figure 3:**
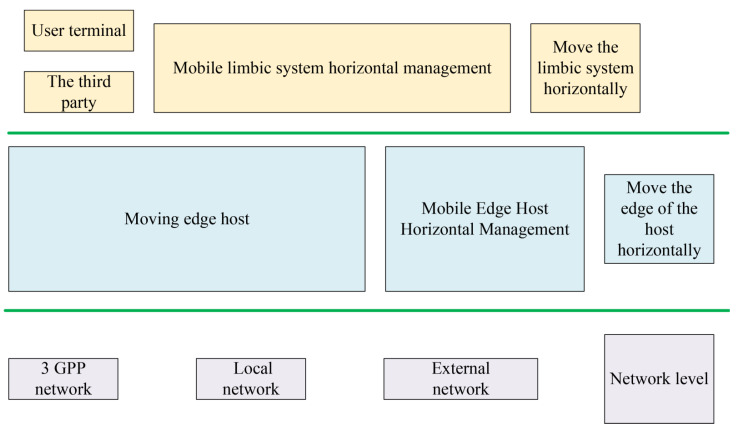
The EC system.

Briefly, the above analysis indicates that EC, as the evolution and development of cloud computing technology, has unique characteristics, such as high applicability, intelligence and flexibility, and highly efficient distribution. Meanwhile, it has a wide application prospect in the field of IoT, industrial manufacturing, and transportation. MEC improves the traditional mobile cloud network, and it computes through the user’s mobile terminals [23,24]. The EC system can be divided into the following three parts, as shown in Figure 3.

### 2.3. Parallel Research on Slope Aspect Algorithm

**Figure 4:**
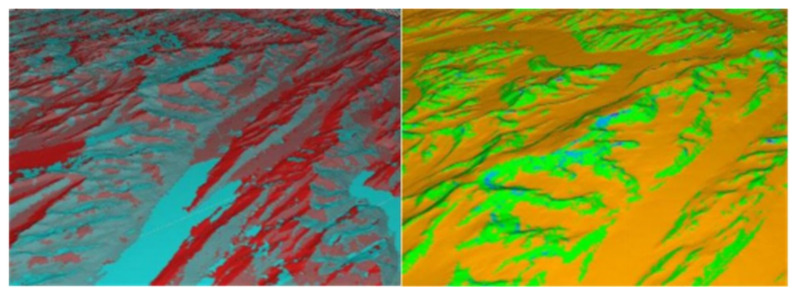
Slope aspect.

Slope aspect algorithm can extract terrain factors from DEM, such as slope factor, aspect factor, broken surface curvature, and partial derivative. The slope factor refers to the gradient of the terrain unit, which is obtained through the arctangent of the ratio of horizontal distance change and height change, and generally uses the percentage unit. Aspect represents the orientation of the terrain unit [25,26]. Figure 4 shows an example of a slope aspect diagram of Xianglan town in Jiamusi city.

In the slope aspect algorithm, the default DEM grid data of GIS [27,28] traverses the image with 3 × 3 window [29] and calculates the value of center A5 with the surrounding values of A1, A4, A6, and A8, and corresponding equations. The slope and slope aspect of A5 can be expressed as in Equations (1) and (2), respectively. In Equations (1) and (2), dx and dy can be expressed as in Equations (3) and (4), and H and V in Equations (3) and (4) can be written as in Equations (5) and (6).

slope = 100 × (dx^2^ + dy^2^)^1/2^ (1)

aspect = tan (dy × dx^−1^) (2)

dx = [(A1 + A4 + A4 + A7) − (A3 + A6 + A6 + A9)] × H ^−1^ (3)

dy = [(A7 + A8 + A8 + A9) − (A1 + A2 + A2 + A3)] × V ^−1^ (4)

H = [(A6 − A4)^2^]^1/2^ × 4 × Z^−1^ (5)

V = [(A2 − A8)^2^]^1/2^ × 4 × Z^−1^ (6)

In Equations (5) and (6), Z represents the elevation scale factor.

The slope aspect algorithm traverses DEM with a 3 × 3 window, so when grid data are divided, all blocks should be extended with a pixel to the upper and lower ends [30,31].

**Figure 5:**
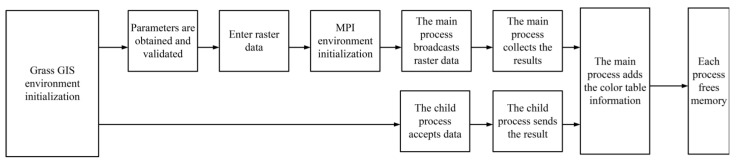
Flowchart of parallel slope aspect algorithm.

The flow diagram of the parallel slope aspect algorithm is shown in Figure 5.

The performances are compared of the parallel task processing architecture and the traditional serial task processing architecture to verify the delay and efficiency of the proposed model for the hydrological spatial analysis. Specifically, the advantages and disadvantages of the traditional serial task processing architecture and the proposed parallel task processing architecture are compared under an increasing number of image pixels. The experiment measures the total time from the beginning of the image pre-processing task to the end of all the related processing tasks of the surrounding edge servers, which is used as the evaluation index. According to the characteristics of the traditional serial task processing architecture, the whole MEC environment has only one edge device and does not support the parallel execution of multiple tasks. By comparison, the proposed parallel task processing architecture contains three edge devices in the MEC environment, and two of them can perform three computing tasks simultaneously, while the other edge device can perform two computing tasks concurrently.

With the improvement of DEM resolution, the calculation amount in the simulation of the hydrological process in the basin increases greatly, which seriously reduces the calculation efficiency of the model. Therefore, serious distortion occurs under watershed terrain information when using low-resolution DEM data, while low efficiency occurs under the calculation of the high-resolution DEM data model. Based on the analysis of the influence of different resolution DEMs on the digital slope characteristics of the WEP-L (Water and Energy Transfer Process in Large River Basin) model, a slope information conversion method from low-resolution DEMs to high-resolution DEMs is proposed through fractal and semivariance function. Generally, the slope of the calculation unit extracted by DEM is the mean slope, and the statistical distribution law of the internal grid elevation is ignored. Since the birth of fractal theory, many studies have found that the natural terrain conforms to the fractal law of different fractal dimensions. Using the method of classification combined with the semivariance function, researchers developed a model for estimating terrain slope under the condition of high-resolution data through low-resolution DEM data, as shown in Equation (7).

S = αd^1−D^ (7)

In Equation (7), S is the regional mean slope of high-resolution DEM, α denotes the coefficients calculated for low-resolution DEM, d stands for the target resolution, D indicates the sub-dimension number of regional grid elevation, and D can be expressed as in Equation (8).

D = a + blnδ(8)

In Equation (8), δ represents the standard deviation of the regional elevation value, a and b are constants.

Here, the regional slope under the condition of low resolution is the determined value, and Equation (9) can be used to calculate the coefficient of low-resolution DEM.

α = S_low_/d^1−D^_low_ (9)

In Equation (9), S_low_ represents the regional mean slope calculated by the same low-resolution DEM, and d_low_ stands for the grid size of low-resolution DEM.

The standard deviation of the grid elevation δ of each sub-basin can be calculated through the statistical analysis function of ArcGIS. According to Equation (9), the slope of the low-resolution DEM region can be converted to that of the high-resolution DEM region. The slope is reclassified according to the slope calculated by the area of each contour zone according to the method of 5 degrees, and the slope area of the corresponding 30 m grid is compared to obtain the best fitting result.

### 2.4. Water Source Pollution Analysis and Treatment Method Based on EC Technology

The experiment base is set on the Xixiaohe Reservoir in Xianglan Town, Tangyuan County, Jiamusi City, and its catchment area is divided into sub-basins using ArcGIS. At the same time, combined with remote sensing technology, the intensity and spatial distribution of pollution sources in each sub-basin were simulated to determine the main pollution sources of each incoming river and the intensity distribution of pollution sources in the catchment area of the convenient reservoir. Then, the actual monitoring value was used to verify the ArcGIS simulation value. Sampling points were set on the inflow rivers of Xixiaohe Reservoir to verify the feasibility and accuracy of ArcGIS simulation results, and the inflow of each pollutant was calculated according to the actual monitoring data. The specific process can be divided into five steps. Step one, flow calculation: according to the precipitation data of the reservoir, the inflow of the reservoir is calculated through the method in the hydrological manual, and then the proportion of each inflow river and the annual inflow of each river are calculated according to ArcGIS hydrological analysis. Step two, sub-basin division: hydrological analysis of DEM data is conducted using ArcGIS, river system distribution in the reservoir catchment area, and catchment area of each reservoir river. Step three, the analysis of land-use types: according to the characteristics of land around the reservoir, the land is classified. Step four, simulation of the distribution of pollution emissions with ArcGIS: pollution emissions are classified and assigned through a land-use map according to land use types, and then they are graded by pollution emission attributes. Step five, the pollution emission is calculated with the vector shear function of ArcGIS in the catchment area of each river in the reservoir, together with the pollution emission and contribution rate of the small basin of each river in the reservoir. Finally, the calculation results of pollution emission distribution are verified.

Here, EC technology was used to analyze and deal with water pollutants. First, EC nodes were set at the key nodes of the water body according to the fixed distance interval. Each EC node collects the control time of the water pollutants in the control area according to the total control node. When the first edge node finds an abnormal pollutant in the water body, the pollutant report is sent to the second edge node in the downstream direction of the water flow. The second EC node sends the pollutant report according to the first EC node. Then, the composition and dose of the pollutant treatment chemicals that need to be put when the pollutant reaches the second EC node are predicted according to the hydrological characteristics and pollutant types. Afterward, the second EC node put pollutant treatment chemicals into the water.

### 2.5. Experimental Environment Configuration and Experimental Data Acquisition

**Figure 6:**
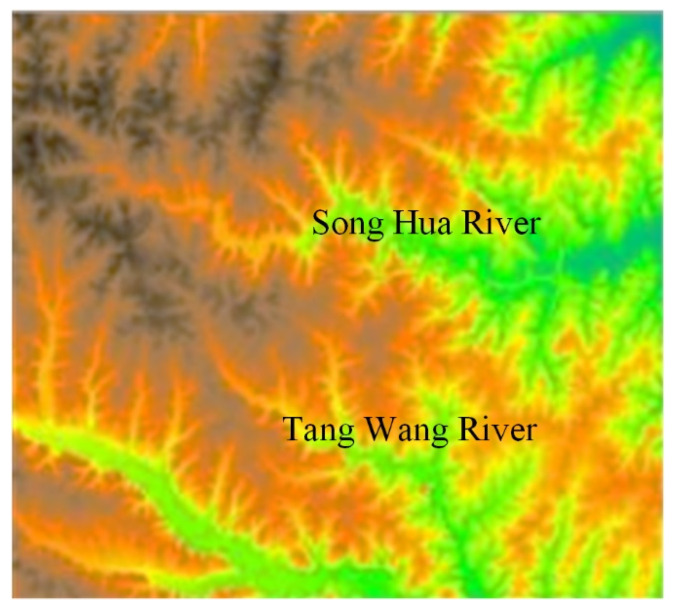
Example of the digital topographic map (30 m).

The experimental environment was configured as follows. The operating system was Ubuntu 18.04, the software is Grass6.4 MPICH, the CPU model was E5506@2.13 GZHz with a frequency of 1600.00. The data from the geographical conditions were used to detect the cloud platform, http://www.dsac.cn/DataProduct/Search?&cateID=10, and the data resolution was 30 m. Figure 6 indicates that the number of rows and columns of the elevation map was 1300 and 1500, respectively, with a total of 195,000 pixels. Meanwhile, the maximum elevation value is 156.32, and the minimum elevation value was 55.67. The projection coordinate system is Lambert conformal conic [32,33].

## 3. Results and Discussion

### 3.1. Algorithm Testing and Simulation

**Figure 7:**
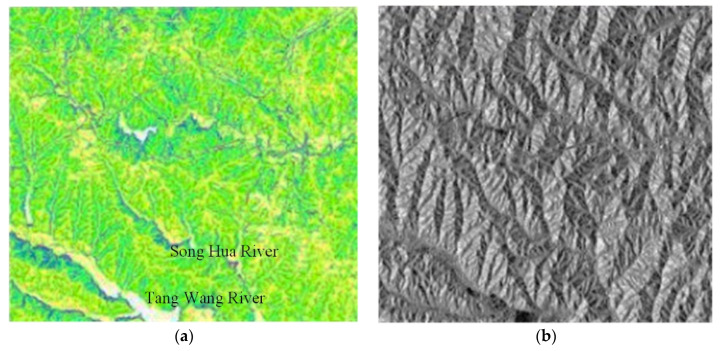
Serial slope aspect algorithm. (**a**, slope graph; **b**, aspect graph).

The slope graph of the serial slope aspect algorithm is shown in Figure 7a, and the aspect graph of the serial slope aspect algorithm is shown in Figure 7b.

**Figure 8:**
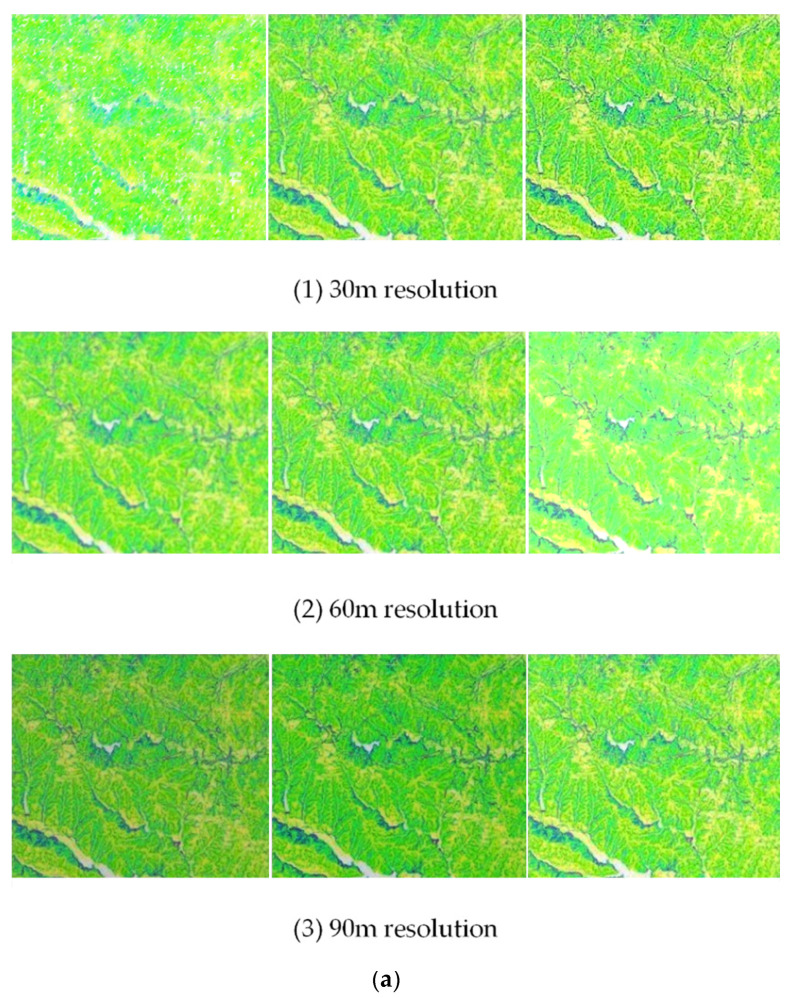

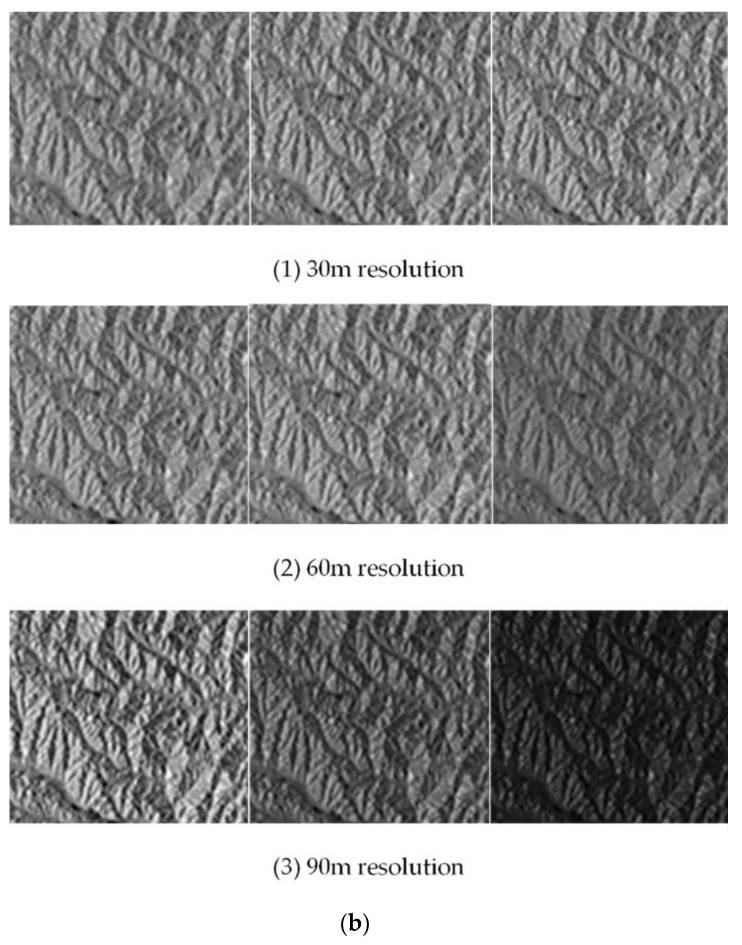
Parallel slope aspect algorithm (**a**, slope graph; **b**, aspect graph).

The slope graph of the parallel slope aspect algorithm is shown in Figure 8a, and the aspect graph of the parallel slope aspect algorithm is shown in Figure 8b.

**Figure 9:**
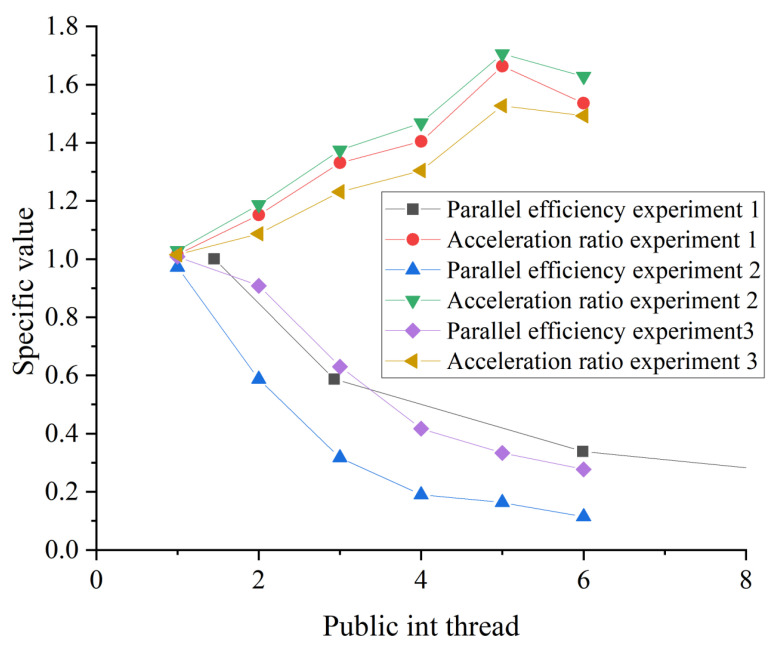
Acceleration ratio and parallel efficiency of parallel slope aspect algorithm.

The experimental results of acceleration ratio and parallel efficiency of parallel slope aspect algorithm are shown in Figure 9**.**

Comprehensive analysis of Figure 7, Figure 8 and Figure 9 suggests that the experimental results of the serial algorithm and parallel algorithm were the same, and the most basic conditions of parallel were satisfied. When the number of processes is 1 to 6, the number of parallel processes is increasing, and the parallel acceleration ratio was also increasing. When the number of processes was greater than 6, the parallel acceleration ratio was decreasing. Overall, because the operation mechanism of the slope aspect algorithm was relatively simple, when the amount of data was small, the advantages of a parallel algorithm were not reflected. Yet, with the number of data increases, each child process can calculate a part of the data, the calculation time is reduced, and the parallel efficiency is improved.

### 3.2. Analysis of Experimental Results of the Parallel Task Processing Algorithm

**Figure 10:**
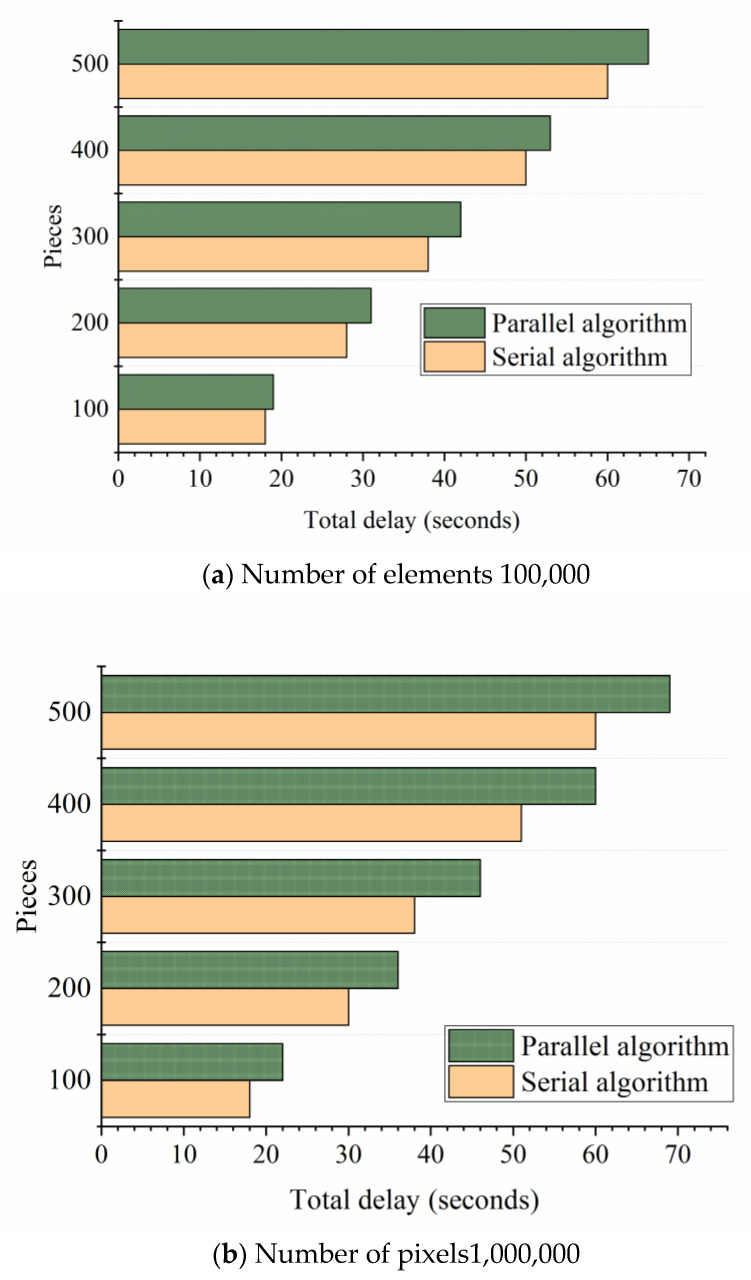

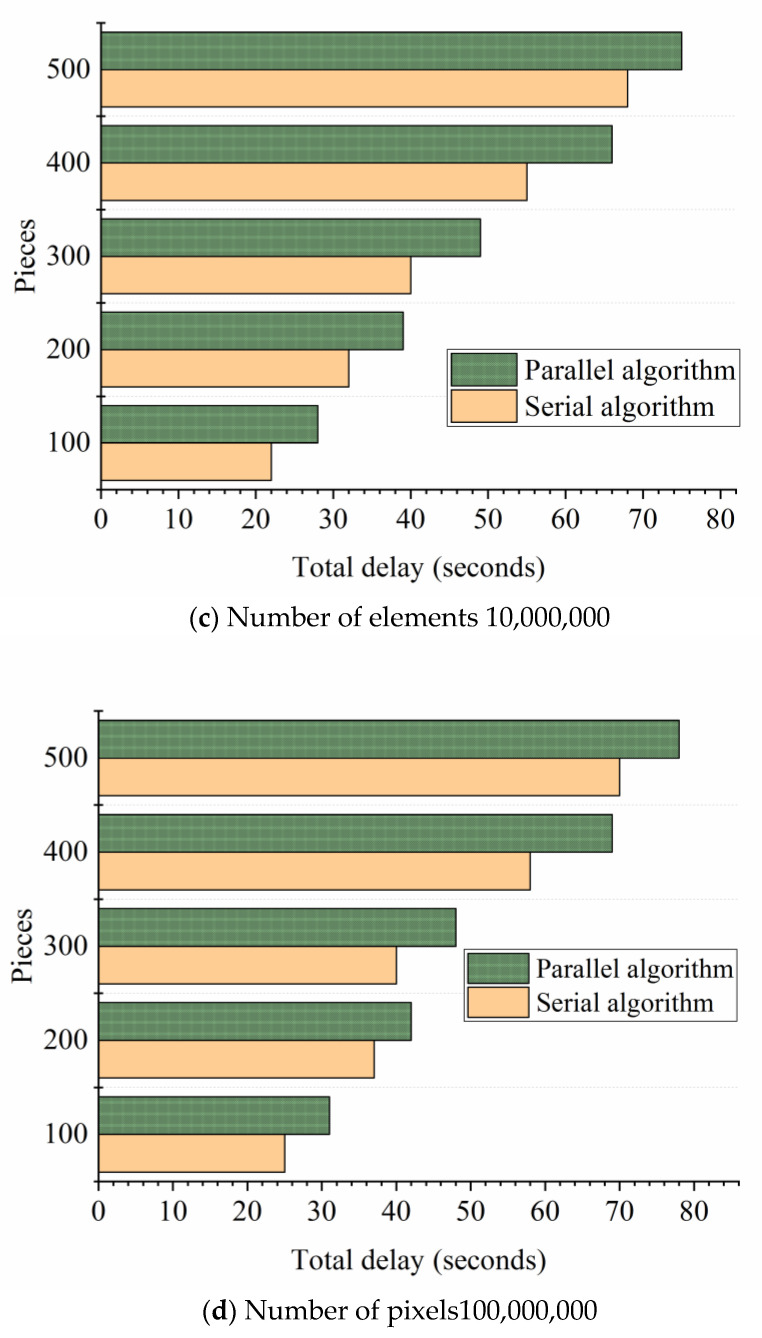
Experimental results of the parallel task processing algorithm.

The comparison results of the average total delay of the proposed parallel task processing algorithm are shown in Figure 10.

Comprehensive experimental results show that the total delay of the proposed parallel task processing framework is less than that of the serial task processing framework. In terms of pixel numbers, the amount of image data with fewer pixels was small. The total delay of the proposed parallel algorithm was similar to that of the serial algorithm. With the increasing amount of data, the advantage of the proposed parallel algorithm was more prominent. Compared with the serial task processing framework, the proposed parallel task processing framework can effectively reduce the total processing delay of many pictures, and the effect is more significant when the frame number and resolution of the video stream were high.

### 3.3. An Empirical Analysis of Ecological Planning

**Figure 11:**
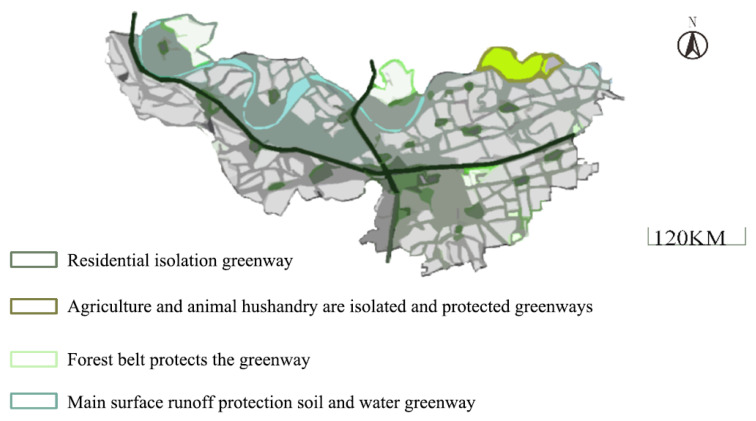
Patch planning of the town’s landscape.

Figure 11 shows the patch planning of the Xianglan town’s landscape, which is taken as an example to analyze its hydrological space and ecological planning.

Figure 11 demonstrates that greenways are set in the periphery of agricultural land, forest land, pastoral area, and residential area to isolate and protect different patches and reduce the fragmentation of the fringe of landscape patches. Significantly, the original forest land should be retained, the overall shape of forest land patches should be ensured, early logging of forest land should be avoided, logging area in forest land should be delimited, and, finally, forest land patches should be formed to maintain ecological stability and sustainable ecological balance. Meanwhile, isolated shelterbelts should be established on both sides of the river to reduce the fragmentation of water margins and protect water and soil. Through the construction of residential areas, pastoral areas, woodlands, green barriers around waters, public green parks, and ecological protection forests, green patches will be formed in the periphery of construction land in these broken areas to reduce the degree of landscape fragmentation and act as an ecological barrier to protect regional ecological balance.

**Figure 12:**
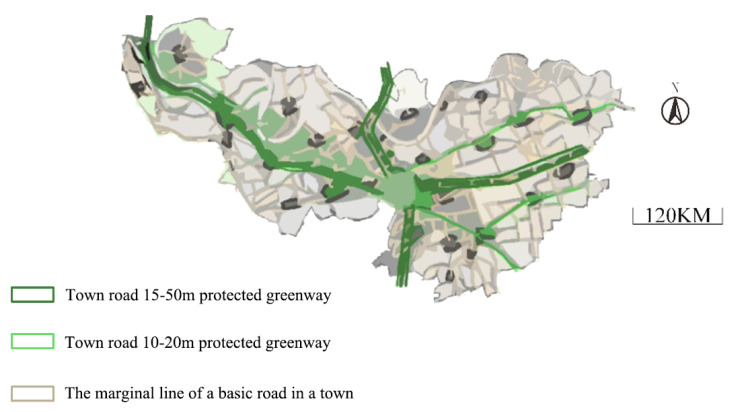
Corridor planning of the town’s landscape.

The corridor planning of the land landscape in town X is shown in Figure 12. The road protection greenways corresponding to different road levels are different. The green circle area in the map is the intersection of the protective forest belt.

The patch landscape of the town is highly fragmented, so the belt corridor can connect the remaining green patches, making it an ecological transportation green corridor in the urban living area. In the original forest land, farmland, pasture, water area, traffic land, and residential area, an isolated green belt is established to form a landscape buffer zone, thereby improving the stability and the ecological environment of the landscape. The traffic land of the town is an artificial corridor, which is linear and acts as a channel and barrier. The number of corridors in traffic land is too large and complex, the fringe shape is irregular, the interference degree is large, and the landscape distribution is unbalanced, which needs to be solved in further landscape planning. The landscape patch traffic land area is also small, so it can be combined with a landscape-type patch landscape area. Additionally, the largest entertainment and sports park can be built in the forest landscape to increase the patch area land of traffic, reduce the complexity of forest patch fringe shape, improve the transportation capacity of the whole town, and promote trade.

### 3.4. The Slope Composition Conversion of Basin Contour Zone Extracted by DEM with Different Resolutions

**Figure 13:**
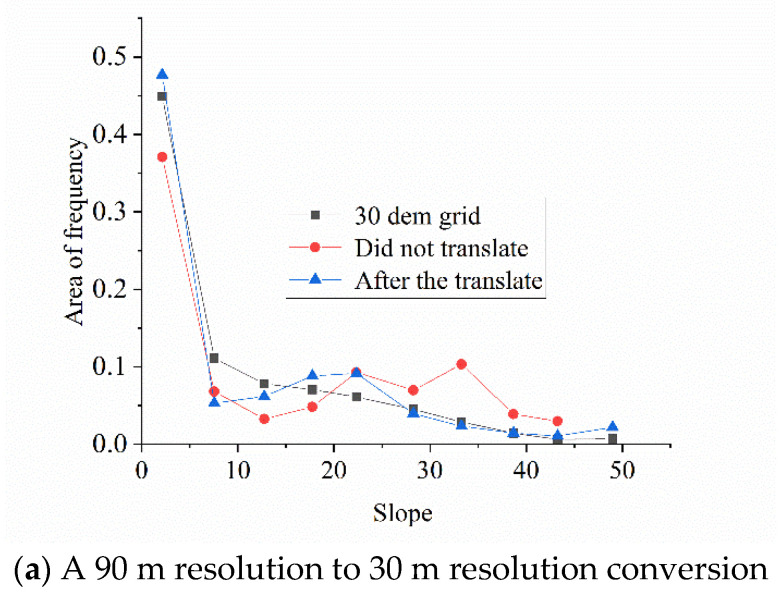

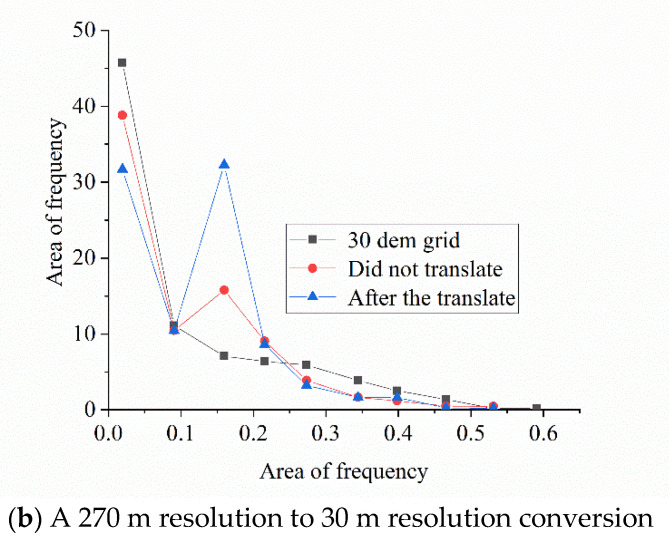
Slope conversion of different resolution DEMs in contour zone.

Figure 13 shows the conversion diagram of different resolution DEMs.

Figure 13a,b implies that the contour slope values extracted from DEM data with different resolutions on different scales are in good agreement with the slope composition of grid area extracted from 30 m DEM data after conversion, indicating that this method effectively can convert the contour slope composition extracted from the low-resolution DEM to the real terrain slope composition, thereby improving the ability of the low-resolution DEM platform to describe terrain after the sub-basin coding.

### 3.5. Verification of Pollutant Emission Distribution Calculation Results

**Figure 14:**
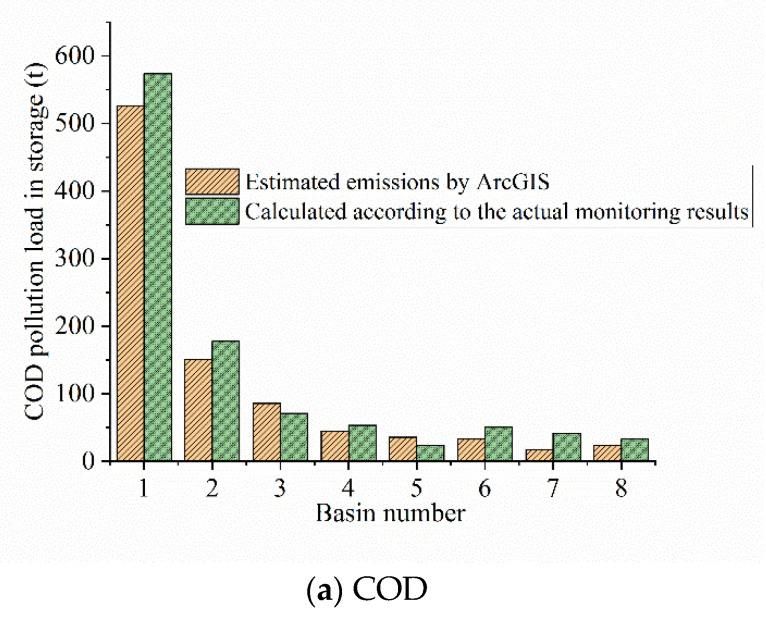

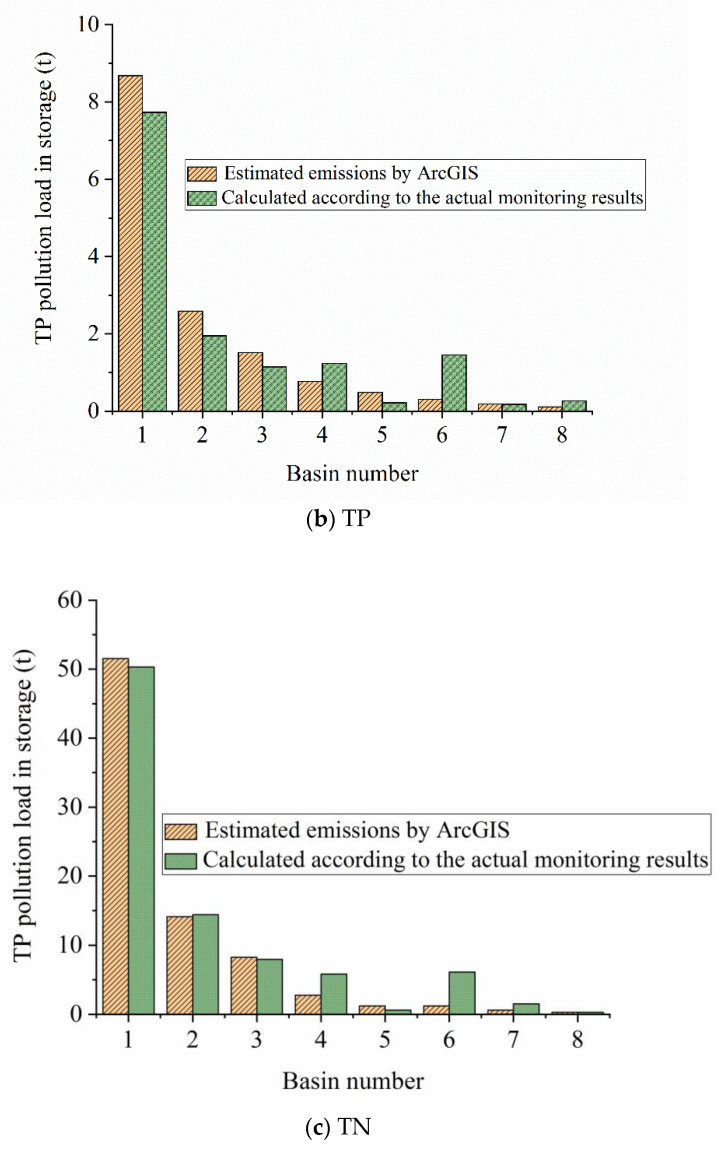
Comparison of inflow pollution load under different algorithms.

Figure 14 shows the comparison of COD (chemical oxygen demand), TP (total phosphorus), and TN (total nitrogen) human pool under different algorithms.

The comparison of the contribution rates of the inflow rivers is shown in Figure 14. The results indicate that compared with the actual monitoring data, the inflow pollution load calculated by the total amount of pollution emissions was in good agreement with that of the inflow rivers. Only the results of several rivers with small contribution rates were slightly different, but the difference was not large. The COD, TN, and TP calculated by the total amount of pollution emissions are 9, 8, and 6% different from the actual monitoring data, respectively.

Innovatively, compared with other research schemes, here, the EC technology was applied to analyze and process water pollutants. Meanwhile, water pollutants were analyzed using ArcGIS technology to divide the catchment area into sub-basins, and then the intensity and spatial distribution of pollution sources in each sub-basin were simulated with remote sensing technology. Finally, the actual monitoring data were used to verify the simulation value of ArcGIS. In water pollutants treatment, the EC technology was used to predict the composition and dose of pollutants treatment agents, which were corrected through the machine learning algorithm until the optimal values were obtained.

## 4. Conclusions

Hydrologic analysis is an important component of the geographic information system, which is often used in regional planning and disaster prediction. The current hydrologic analysis algorithms have the disadvantages of a long time consuming and low accuracy in data processing, and most of the existing hydrologic analysis algorithms are serial algorithms. Therefore, EC is used to improve the slope aspect algorithm in hydrologic analysis. First, analyze the existing hydrological analysis algorithms and study their applicability. Second, analyze the slope aspect algorithm principle and implement it on the corresponding platform. Third, realize the parallel slope aspect algorithm architecture by using EC. Fourth, use ArcGIS technology to simulate the inflow of pollutants. In addition, EC technology is also used to predict the concentration of chemicals needed to remove pollutants from the reservoir. However, there are still some shortcomings. The study designs the algorithm only when the process number 1 ~ 6 can improve the speed ratio. When the number of processes is greater than 6, the parallel efficiency decreases. How to keep the speedup ratio stable when the number of processes is increased needs further exploration.

## Data Availability

Not applicable.
